# Supplementary material for: Improving the Long-Term Mechanical Properties of Thermoplastic Short Natural Fiber Compounds by Using Alternative Matrices
Source: Biomimetics (Basel). 2025 Jan 13;10(1):46. doi: 10.3390/biomimetics10010046 (PMC11761199; doi:10.3390/biomimetics10010046)
Supplement: Supplementary file 1 [file biomimetics-10-00046-s001.zip › biomimetics-3327081-supplementary.pdf]

Supplementary materials.

Improving the long-term mechanical properties of thermoplastic short natural fiber compounds by using alternative matrices

Renato Lemos Cosse [1], Tobias van der Most [1], Vincent Voet [1] , Rudy Folkersma [1] and Katja Loos [2,*]

S1: Optical micrographs of wood and glass fibers

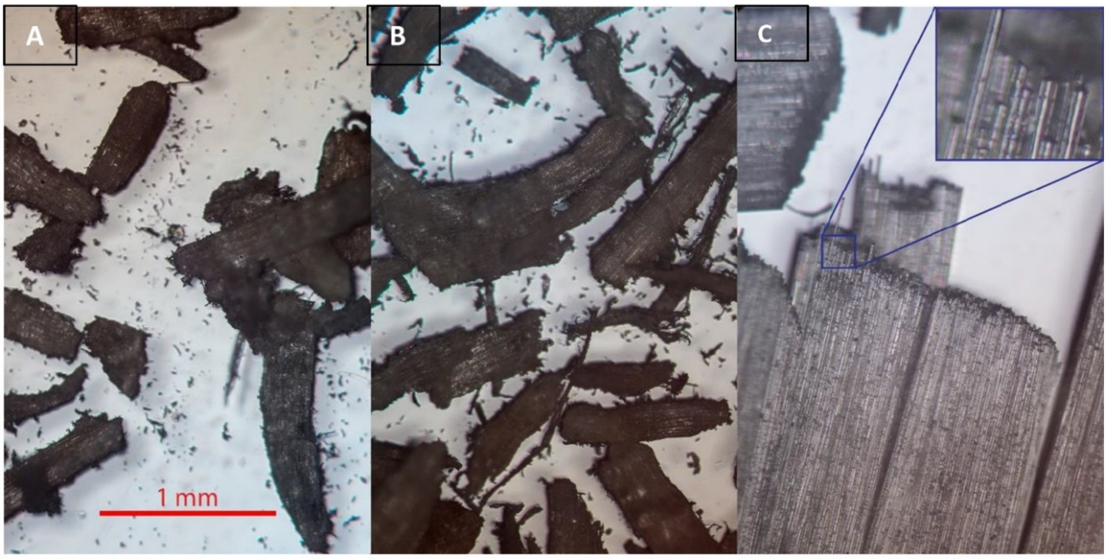

Figure S1. Optical micrographs of wood and glass fibers. Wood and glass fiber prior to compounding. (A) C320, (B) C400, (C) glass fiber.

S2: Schematic of the grafting reaction through reactive extrusion

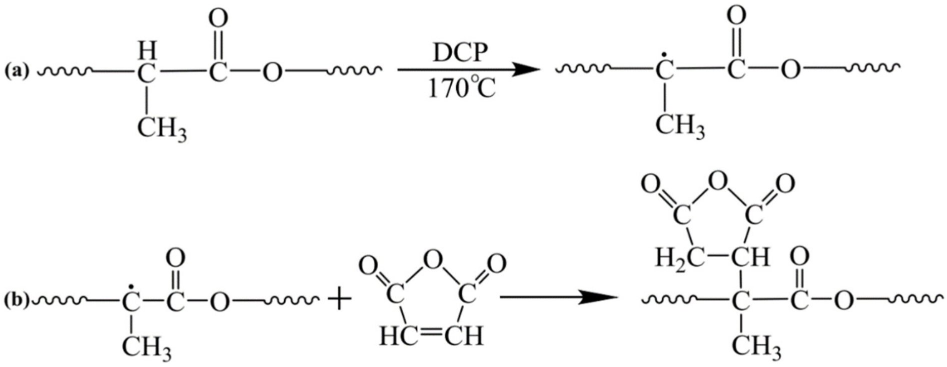

Figure S2. Schematic diagram of PLA grafted with maleic anhydride. At a heightened temperature, peroxide decomposes and starts the reaction of grafting MA onto PLA chains [33].

S3: Schematic of the twin-screw extruder configuration

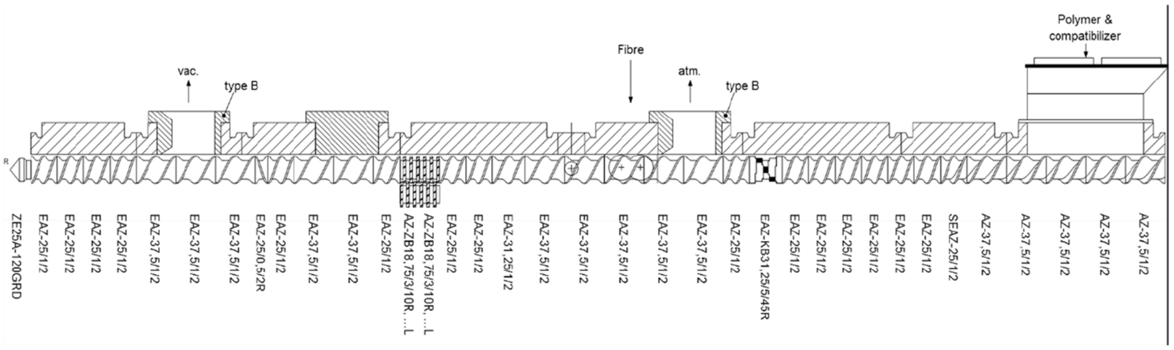

Figure S3. Barrel and screw configuration of the twin-screw corotating compounder (extruder). The polymer and compatibilizer are fed into the hopper (on the right), and they are transported forward until they reach the kneading element, in which the tooth direction is in the counter turning direction, ensuring that all the polymer material is molten afterwards. By using a larger pitch, the melt is pulled apart to create space to allow fibers to be added with a side feeder (ø25 mm). The material is then kneaded by forward transport, mixed with mixing elements (tooth blocks), transported forward, temporarily slowed down with a retaining element (to create a seal) for vacuum degassing, vacuum degassed and then extruded

S4: Pellets and their respective injection molded samples

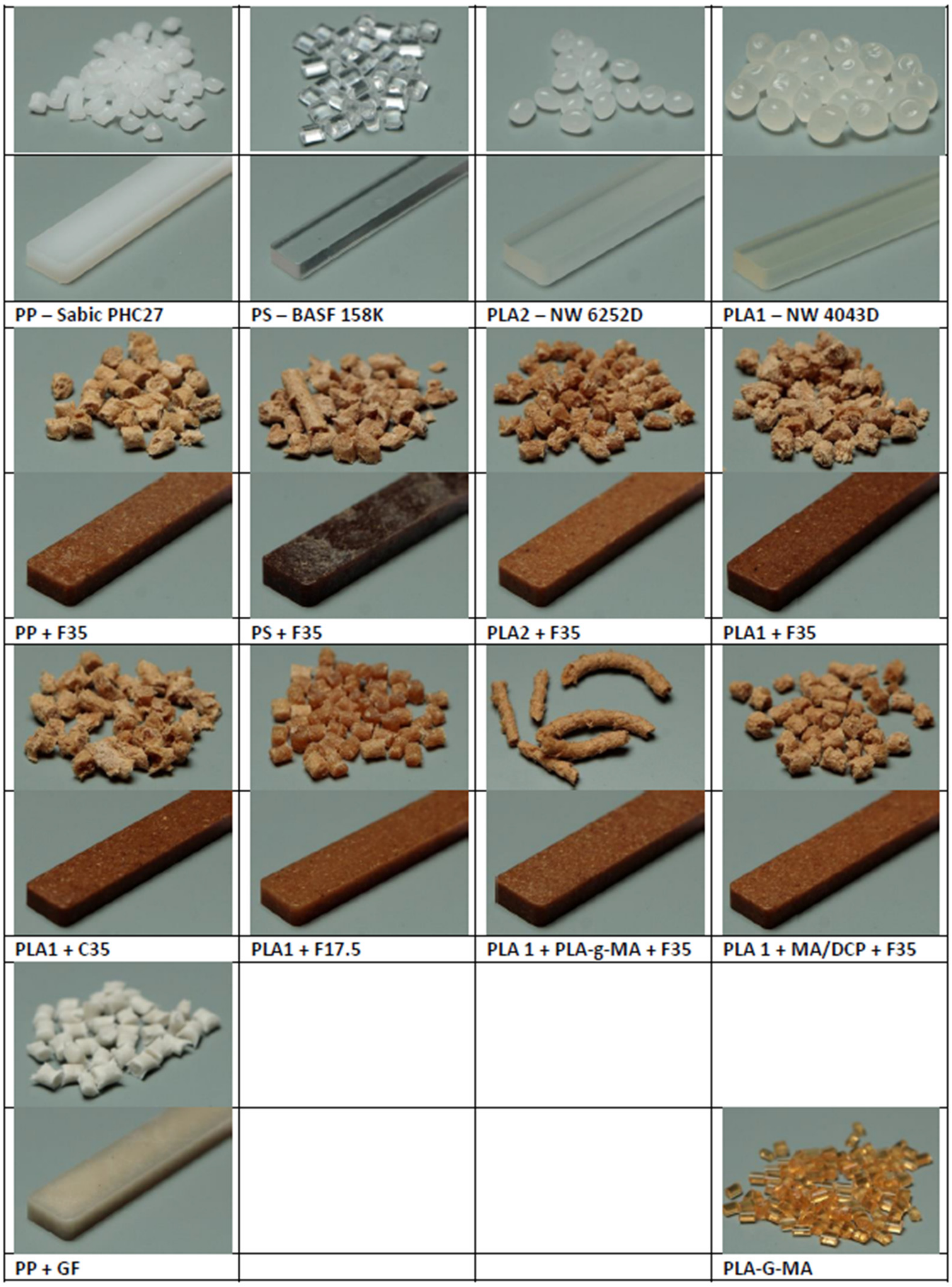

Figure S4. Pellets and injection molded bar colors

S5:Tensile and Flexural test results of pure polymer and their compatibilized version

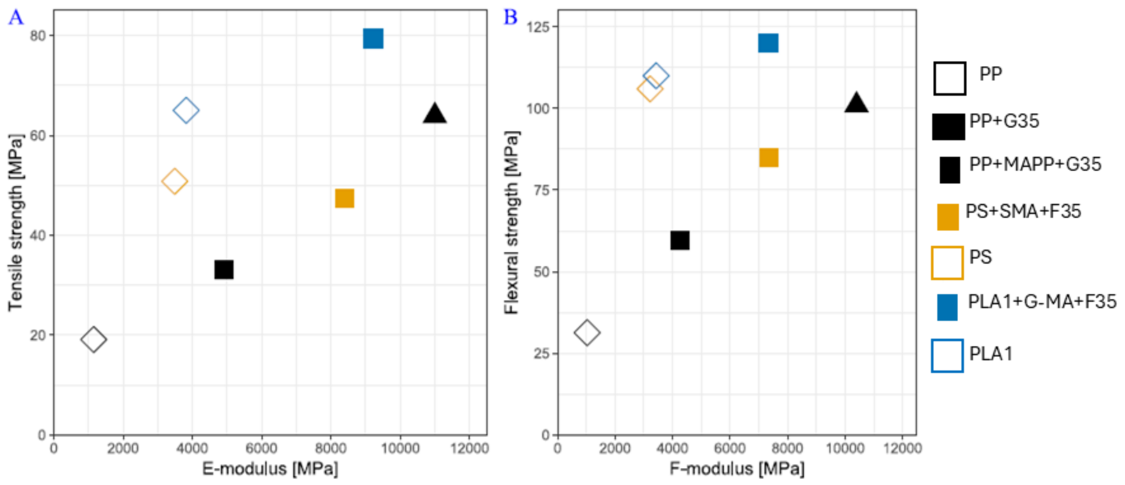

Figure S5. Tensile (A) and flexural (B) test results of pure polymers and their compatibilized versions.

S6: Tensile and Flexural test results of crystallized PLA and its composites.

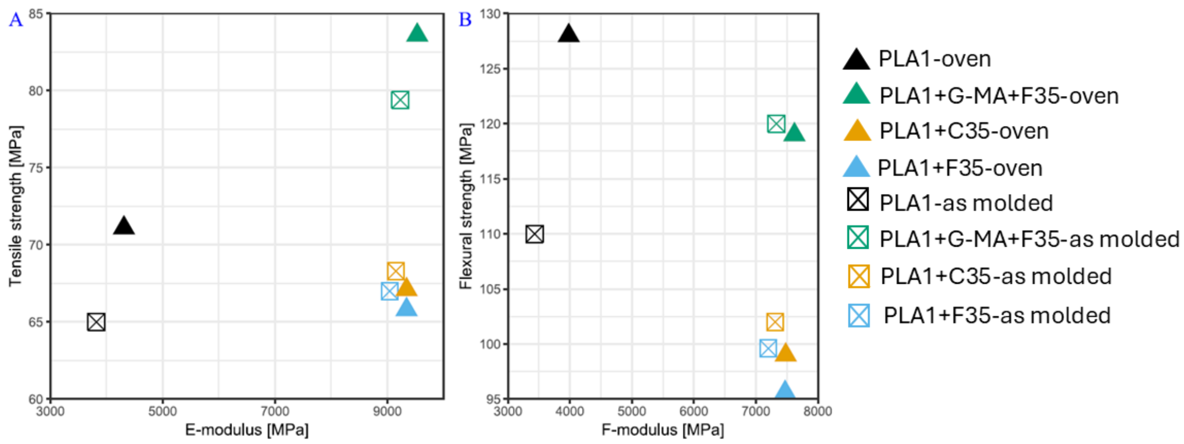

Figure S6. Tensile (A) and Flexural (B) test results of crystallized PLA and its composites.

S7: Granulometric range of wood fibers used for the reinforcement of plastic wood composites

Table S1. Screen analysis of the wood fiber reinforcement. The test results were retrieved from the supplier (J. Rettenmaier & Söhne GMBH).

	Arbocel C320 (F)	Arbocel C400 (C)
> 100 µm	95.3 %	92.4 %
> 250 µm	32.1 %	57.1 %
> 400 µm	< 0.01 %	16.7 %

S8: Moisture Absorption

Table S2. Mass changes in pure polymers and their respective composites after being submerged for hours in water.

Hours soaking	24	48	72	96	168	264	360	504	672
PP	0,013	0,012	0,023	0,033	0,043	0,116	0,019	0,040	0,027
PP+GF	0,041	0,047	0,111	0,153	0,095	0,114	0,172	0,134	0,152
PP+F35	0,538	0,707	0,868	0,924	1,144	1,418	1,553	1,802	2,006
PS	0,078	0,072	0,100	0,130	0,048	0,024	0,056	0,024	0,036
PS+F35	0,893	1,135	1,478	1,736	2,128	2,666	3,163	3,707	4,325
PLA1	0,216	0,263	0,365	0,381	0,455	0,508	0,560	0,584	0,582
PLA2	0,147	0,239	0,288	0,309	0,400	0,475	0,549	0,536	0,582
PLA1+F35	1,260	1,685	2,184	2,503	3,227	4,049	4,820	5,706	6,548
PLA1+C35	1,251	1,767	2,175	2,522	3,290	4,152	4,856	5,843	6,687
PLA1+F17,5	0,665	0,871	1,105	1,342	1,654	2,066	2,409	2,814	3,175
PLA1+GMA +F35	1,259	1,803	2,186	2,568	3,331	4,188	4,939	5,980	6,781
PLA1+MA +F35	1,171	1,650	2,112	2,415	3,193	4,000	4,749	5,658	6,506
PLA2+F35	1,333	1,898	2,372	2,732	3,590	4,585	5,435	6,541	7,803

S9: Tensile modulus after exposure to various moisture conditions.

Table S3 Tensile modulus of the polymers and their respective composites subjected to different environmental conditions. where “x” represents the mean and “s” represents the standard deviation.

E-modulus [MPa]	x 10%	s 10%	x 50%	s 50%	x 90%	s 90%	x soak	s soak	x SFT	s SFT
PP	1160	6	1150	10	1180	13	1180	6	1170	12
PP+G35	11700	131	11000	180	11300	177	11400	61	11300	138
PP+MAPP+F35	5000	37	4910	33	4850	43	4680	24	4900	19
PS	3510	28	3490	15	3510	11	3510	21	3510	15
PS+SMA+F35	8540	96	8400	88	7630	13	6980	50	7900	31
PLA1	3940	25	3820	20	3730	27	3750	14	3830	23
PLA2	3910	21	3820	24	3730	13	3750	28	3840	35
PLA1+F35	9180	89	9040	82	7240	47	6320	57	7660	124
PLA1+C35	9170	21	9150	185	7400	41	6340	34	7610	91
PLA1+F17.5	6280	21	6390	138	5490	63	5190	41	5720	27
PLA1+G-MA +F35	9370	46	9230	88	7470	149	6450	74	8090	134
PLA1+MA+DCP +F35	9240	54	9200	188	7400	57	6540	69	7970	66
PLA2+F35	9250	48	9110	71	7180	38	5800	34	7300	55

S10: Tensile strength of the polymers and their compounds after exposure to various moisture conditions.

Table S4. Tensile strength of the polymers and their respective composites subjected to different environmental conditions. Where “x” represents the mean and “s” represents the standard deviation.

Strength [MPa]	x 10%	s 10%	x 50%	s 50%	x 90%	s 90%	x soak	s soak	x SFT	s SFT
PP	19,2	0,1	19,1	0,1	19,3	0,1	19,4	0,1	19,4	0
PP+G35	64,7	0,8	63,9	0,8	64,5	0,6	64	0,9	63,4	0,6
PP+MAPP+F35	33,8	0,2	33,1	0,1	32,8	0,0	32,1	0,2	33,1	0
PS	51,2	0,6	50,8	0,9	51,7	0,7	51,7	0,8	50,8	0,2
PS+SMA+F35	50,7	0,6	47,4	1,1	49,4	0,7	47,3	0,3	50,5	1
PLA1	66,1	0,3	65,0	0,7	57,6	0,1	55,3	0,3	62	0,4
PLA2	66,3	0,2	64,2	0,1	58,8	0,2	55,6	0,4	62	0,5
PLA1+F35	68,1	0,5	67,0	0,6	55,1	0,4	48,8	0,3	55,8	0,6
PLA1+C35	70,4	0,4	68,3	0,5	57,2	0,2	49,5	0,2	56,9	0,3
PLA1+F17.5	64,1	0,1	62,8	0,3	53,9	0,1	51,7	0,4	57	0,1
PLA1+G-MA+F35	80,8	0,1	79,4	0,5	64,7	0,6	55,7	0,4	69,8	0,9
PLA1+MA+DCP+F35	80,0	0,1	77,9	0,9	62,5	0,4	55,8	0,3	68,8	0,1
PLA2+F35	66,3	0,2	63,9	0,2	52,8	0,2	43,2	0,1	48,8	0,5

S11: Deformation of polymers and their compounds at maximum tensile strength under various moisture conditions

Table S5. Deformation at maximum tensile strength under various moisture conditions, including 10%, 50% and 90% relative humidity and freeze–thaw cycles. Where "x" represents the mean and "s" represents the standard deviation.

dL at σ-max [%]	x 10%	s 10%	x 50%	s 50%	x 90%	s 90%	x soak	s soak	x SFT	s SFT
PP	6,2	0,1	6,4	0,1	6,2	0,2	6,0	0,1	6,0	0,1
PP+G35	1,0	0,1	1,1	0,2	1,1	0,0	1,0	0,1	1,0	0,0
PP+MAPP+F35	1,8	0,0	1,8	0,0	2,0	0,0	2,0	0,0	1,9	0,0
PS	2,1	0,2	2,1	0,2	2,1	0,1	2,1	0,2	2,3	0,0
PS+SMA+F35	0,7	0,0	0,6	0,0	0,8	0,0	0,9	0,0	0,8	0,0
PLA1	2,2	0,0	2,3	0,0	1,9	0,0	1,8	0,0	2,0	0,0
PLA2	2,1	0,0	2,3	0,0	2,0	0,0	1,8	0,0	2,0	0,0
PLA1+F35	1,0	0,0	1,0	0,0	1,2	0,0	1,3	0,0	1,1	0,0
PLA1+C35	1,0	0,0	1,0	0,0	1,2	0,0	1,3	0,0	1,1	0,0
PLA1+F17.5	1,5	0,0	1,5	0,0	1,5	0,0	1,6	0,0	1,5	0,0
PLA1+G-MA+F35	1,3	0,0	1,3	0,0	1,5	0,0	1,6	0,1	1,4	0,0
PLA1+MA+DCP+F35	1,3	0,0	1,3	0,0	1,5	0,0	1,6	0,0	1,3	0,0
PLA2+F35	0,9	0,0	0,9	0,0	1,1	0,0	1,2	0,0	1,0	0,0

S12: Flexural creep of pure polymers and their compatibilized compounds.

Table S6. Flexural modulus of the pure polymers and their compounds at different working temperatures.

	F-modulus	load	initial deformation 20°C	2 hr. creep deformation 20°C	total deformation 20°C	creep /initial 20°C	initial deformation 40°C	2 hr. creep deformation 40°C	total deformation 40°C	creep /initial 40°C	initial deformation 60°C	2 hr. creep deformation 60°C	total deformation 60°C	creep /initial 60°C
	MPa	MPa	%	%	%	%	%	%	%	%	%	%	%	%
PP	1030	2,060	0,156	0,114	0,270	73,077	0,198	0,402	0,600	203,030	0,294	0,888	1,181	302,041
PP+GF	10400	20,800	0,192	0,060	0,252	31,250	0,216	0,189	0,405	87,500	0,261	0,417	0,678	159,770
PP+F35	4250	8,500	0,186	0,069	0,255	37,097	0,204	0,231 %	0,435	113,235	0,276	0,489	0,765	177,174
PS	3220	6,440	0,187	0,012	0,199	6,452	0,196	0,030	0,226	15,385	0,199	0,328	0,527	165,152
PS+F35	7360	14,720	0,200	0,018	0,218	9,091	0,203	0,057	0,260	28,358	0,215	0,278	0,493	129,577
PLA1	3430	6,86	0,199	0,021	0,220	10,606	0,202	0,377	0,579	186,567	-	-	-	-
PLA1+GMA+F35	7330	14,66	0,193	0,015	0,208	7,813	0,187	0,175	0,362	93,548	-	-	-	-

S13: Flexural modulus of PLA and various compounds.

Table S7. Flexural creep behavior of PLA and its compounds at different work temperatures.

	F-modulus	load	initial deformation 20°C	2 hr. creep deformation 20°C	total deformation 20°C	creep/initial 20°C	initial deformation 40°C	2 hr. creep deformation 40°C	total deformation 40°C	creep/initial 40°C
	MPa	MPa	%	%	%	%	%	%	%	%
PLA1	3430	6,86	0,199	0,021	0,220	10,606	0,202	0,377	0,579	186,567
PLA2	3420	6,84	0,186	0,024	0,211	12,903	0,195	0,406	0,601	207,692
PLA1+F35	7200	14,40	0,190	0,018	0,208	9,524	0,202	0,202	0,404	100,000
PLA1+C35	7310	14,62	0,196	0,012	0,209	6,154	0,199	0,187	0,387	93,939
PLA1+F17.5	5430	10,86	0,190	0,015	0,205	7,937	0,199	0,235	0,434	118,182
PLA1+G-MA+ F35	7330	14,66	0,193	0,015	0,208	7,813	0,187	0,175	0,362	93,548
PLA1+MA+ DCP+F35	7370	14,74	0,196	0,018	0,215	9,231	0,202	0,196	0,399	97,015
PLA2+F35	7350	14,70	0,202	0,018	0,221	8,955	0,202	0,190	0,393	94,030
